# Controlling Andreev Bound States with the Magnetic Vector Potential

**DOI:** 10.1021/acs.nanolett.2c03130

**Published:** 2022-10-24

**Authors:** Christian M. Moehle, Prasanna K. Rout, Nayan A. Jainandunsing, Dibyendu Kuiri, Chung Ting Ke, Di Xiao, Candice Thomas, Michael J. Manfra, Michał P. Nowak, Srijit Goswami

**Affiliations:** † QuTech and Kavli Institute of Nanoscience, Delft University of Technology, 2600 GADelft, The Netherlands; ‡ Academic Centre for Materials and Nanotechnology, AGH University of Science and Technology, 30-059Krakow, Poland; § Department of Physics and Astronomy, Purdue University, West Lafayette, Indiana47907, United States; □ Elmore School of Electrical and Computer Engineering, Purdue University, West Lafayette, Indiana47907, United States; ○ School of Materials Engineering, Purdue University, West Lafayette, Indiana47907, United States; △ Microsoft Quantum Lab West Lafayette, West Lafayette, Indiana47907, United States

**Keywords:** Planar Josephson junctions, Tunneling spectroscopy, Andreev bound states, Local superconducting phase difference

## Abstract

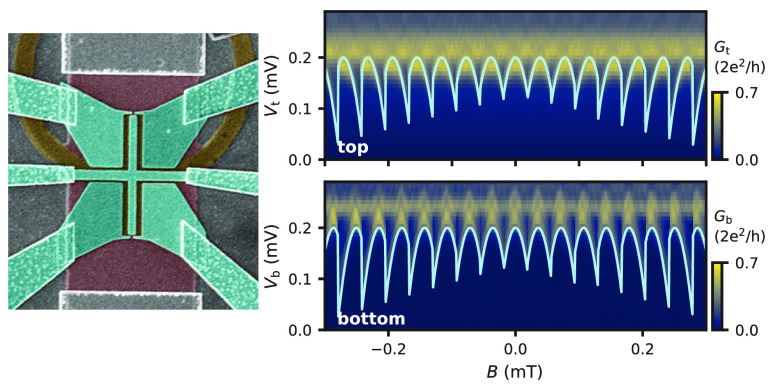

Tunneling spectroscopy measurements are often used to probe the energy spectrum of Andreev bound states (ABSs) in semiconductor-superconductor hybrids. Recently, this spectroscopy technique has been incorporated into planar Josephson junctions (JJs) formed in two-dimensional electron gases, a potential platform to engineer phase-controlled topological superconductivity. Here, we perform ABS spectroscopy at the two ends of planar JJs and study the effects of the magnetic vector potential on the ABS spectrum. We show that the local superconducting phase difference arising from the vector potential is equal in magnitude and opposite in sign at the two ends, in agreement with a model that assumes localized ABSs near the tunnel barriers. Complemented with microscopic simulations, our experiments demonstrate that the local phase difference can be used to estimate the relative position of localized ABSs separated by a few hundred nanometers.

Hybrid structures composed of superconductors and normal conductors host Andreev bound states (ABSs).^1−3^ These states are superpositions of electron-like and hole-like excitations with energies lower than the superconducting gap. In recent years, superconductor–semiconductor hybrids have emerged as an appealing platform to manipulate these bound states. For example, controllable coupling between individual ABSs has led to the creation of Andreev molecules,^4−7^ and Josephson junctions (JJs) based on these hybrids have been combined with superconducting circuits to realize Andreev qubits.^8,9^ In JJs, the microscopic properties of ABSs determine global properties of the junction, such as its critical current.^2^ The energy of ABSs is dependent on the phase difference between the superconducting leads, which can be tuned by the application of a magnetic flux through a superconducting loop connecting the leads. In planar JJs, the vector potential of the magnetic field leads to streams of positive and negative current, to the formation of Josephson vortices, and to the well-known Fraunhofer interference pattern in critical current.^10−12^ It has been proposed that such planar JJs can host Majorana bound states,^13−16^ and that the location and coupling of these states can be controlled via the vector potential.^17^

In order to investigate how the vector potential modifies ABSs in a JJ, one needs experimental techniques that provide information about the spatial extent and location of ABSs. Studies in junctions that simultaneously probe the spatial distribution and energy spectrum of ABSs have mainly been performed using scanning probe techniques,^18,19^ and more recently, via local tunnel probes in two-dimensional electron gases (2DEGs).^20,21^

Here, we perform tunneling spectroscopy at both ends of planar JJs embedded in a superconducting loop, allowing us to probe the effects of the magnetic vector potential on the phase-dependence of the ABS energy. We directly show that the local superconducting phase difference originating from the vector potential has equal magnitude but opposite sign at the two ends of the JJ. This is manifested by a striking difference in the spectroscopy maps obtained from each side, in excellent agreement with a model that assumes tunnel coupling to a single ABS localized at each end. Microscopic numerical simulations confirm that such a localization of the ABSs is indeed expected, and that the tunneling current is only sensitive to ABSs located near the ends of the JJ. By modifying the potential landscape in the vicinity of the tunnel probe, we show that the local phase difference allows us to resolve multiple ABSs within a spatial extent of a few hundred nanometers, in qualitative agreement with simulations.

The JJs are fabricated using an InSb_0.92_As_0.08_ 2DEG with in situ grown Al as the superconductor (details about the molecular beam epitaxy growth of the heterostructure can be found in ref (22)). Figure 1a shows a schematic and a false-colored scanning electron micrograph (SEM) of such a device. To fabricate the devices, we first use a combination of Al and 2DEG etches to define the JJ and the superconducting loop. The exposed 2DEG on the top and bottom sides of the JJ is contacted by Ti/Au, and the Al loop is contacted by NbTiN, resulting in a three-terminal device. A globally deposited layer of AlO_*x*_ forms the gate dielectric. Lastly, split gates are evaporated on the top and bottom ends of the JJ, allowing us to define tunnel barriers, while also depleting the 2DEG around the junction. A central gate (kept grounded throughout this study) covers the normal section of the JJ. We study two JJs (Dev 1 and Dev 2), both with length *l* = 80 nm and width *w* = 5 μm. More details about the device fabrication can be found in the Supporting Information - Section 1 (SI-1). The devices are measured in a dilution refrigerator with a base temperature of 30 mK using standard lock-in techniques.

**Figure 1:**
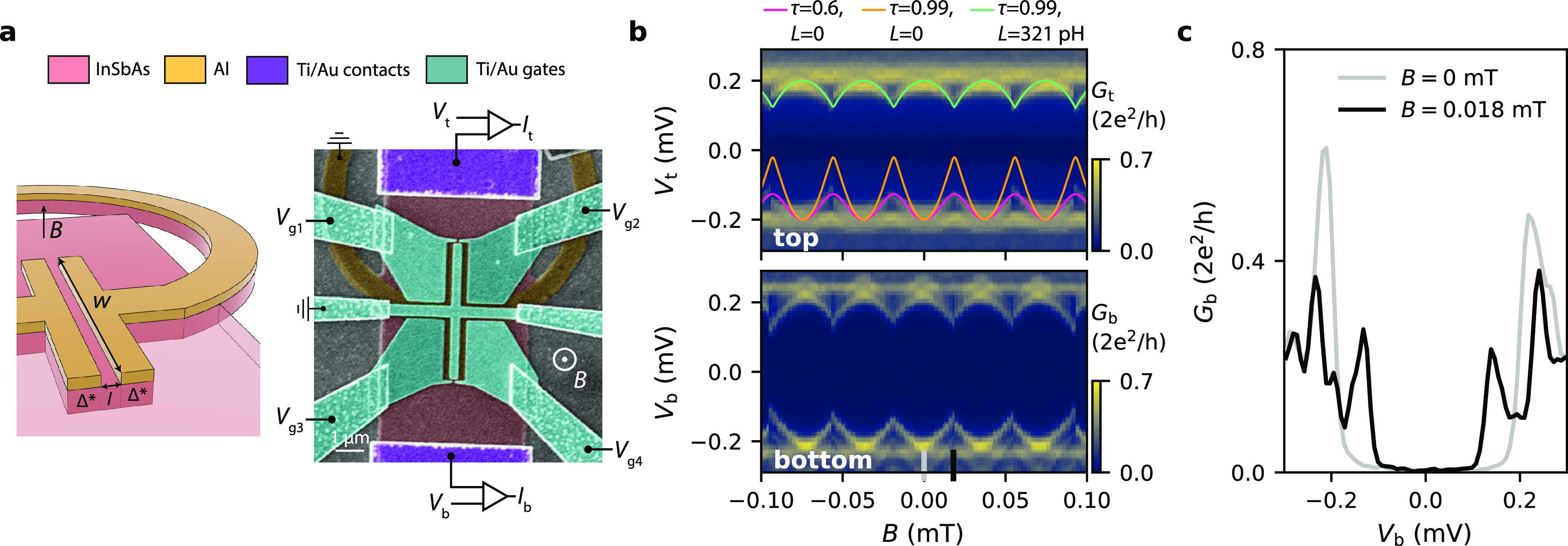
Tunneling spectroscopy at the two ends of a planar phase-biased JJ. (a) Schematic (before the gate deposition) and false-colored SEM of Dev 1. (b) Spectroscopy maps measured at the top (*V*_g1_ = −0.39 V, *V*_g2_ = −0.74 V, *V*_g3_ = 0 V, *V*_g4_ = 0 V) and bottom end of the JJ (*V*_g1_ = 0 V, *V*_g2_ = 0 V, *V*_g3_ = −1.1 V, *V*_g4_ = −0.6 V). The three curves in the top panel correspond to single-channel ABS spectra calculated for different combinations of transmission (τ) and loop inductance (*L*) as specified in the legend. (c) Line cuts of the bottom spectroscopy map at the indicated positions in panel b.

In Figure 1b (top panel) we present a tunneling spectroscopy map for Dev 1 at the top end of the JJ. The conductance, *G*_t_ = d*I*_t_/d*V*_t_, is measured as a function of voltage bias, *V*_*t*_, and perpendicular magnetic field, *B*. The bottom panel shows the conductance measured at the bottom end, *G*_b_ = d*I*_b_/d*V*_b_, with representative line cuts presented in Figure 1c. In both maps we see a superconducting gap that is modulated by *B*, with an oscillation period equal to Φ_0_/*S*, where Φ_0_ = *h*/2*e* is the magnetic flux quantum and *S* is the area of the superconducting loop. This modulation indicates the presence of flux-periodic ABSs in the JJ. For a normal region much shorter than the superconducting coherence length, the relation between the ABS energy and the gauge-invariant phase difference between the two superconducting leads, φ, is given by^2^
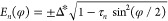
1where Δ* is the induced gap in the 2DEG regions below the Al leads and τ_*n*_ is the transmission probability of the *n*th conduction channel. The flux through the loop, Φ = *BS*, and φ are related via φ = 2πΦ/Φ_0_. The relatively small modulation depth observed in the experiment might suggest low-transmission ABSs [see the field evolution of a single ABS with τ = 0.6 (pink) and τ = 0.99 (orange) in Figure 1b]. However, when looking more closely at the energy minima, we find that they display pronounced cusps, not expected from eq 1. These cusps are indicative of phase slips that occur when the superconducting loop has a sizable inductance, *L*, whereby the standard linear flux-phase relation no longer holds. We independently estimate *L* = 321 pH (see SI-2) and use the appropriate flux-phase conversion (see SI-6) to find that the measured ABS spectrum is consistent with a large transparency of τ = 0.99 (light green line in Figure 1b). We further confirm this by performing spectroscopy at higher *B*, as will be discussed later. This highlights the fact that the inductance, which can be significant in thin film superconductors, strongly affects the ABS spectra observed in experiments.

Thus far we have assumed that the superconducting phase difference is constant along the width of the JJ (see Figure 2a for a top-view schematic of the junction). However, the vector potential of the magnetic field creates a phase gradient, ϕ′(*y*), and the total gauge-invariant phase difference is given by φ(*y*) = ϕ + ϕ′(*y*), where ϕ is the phase difference that can be tuned by the flux through the loop. The position-dependent local phase difference can be expressed as^10,23^
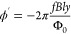
2where *f* is a flux focusing factor that increases the effective magnetic flux in the JJ (see SI-3 andRef. (24)). This expression for φ is valid for JJs with a width much smaller than the Josephson penetration length, which is the case for our junctions (see SI-4). The magnetic vector potential also leads to the formation of localized ABSs with a well-defined supercurrent direction (see SI-7 for numerical simulations). Figure 2b shows a plot of the expected local phase difference for Dev 1 at *B* = 1 mT, demonstrating that the phase difference experienced by an ABS located at the top and bottom end of the JJ will be significantly different. Therefore, for localized ABSs (as depicted in Figure 2a), one expects observable differences in the field evolution of their energies. This is more clearly illustrated in Figure 2c, where we plot the ABS energy, *E*, as a function of *B*. As *B* increases, the maxima for the top and bottom ABS shift relative to each other. This is a direct consequence of eq 2, whereby ABSs located at opposite ends of the JJ are sensitive to the local phase difference with equal magnitude but opposite sign.

**Figure 2:**
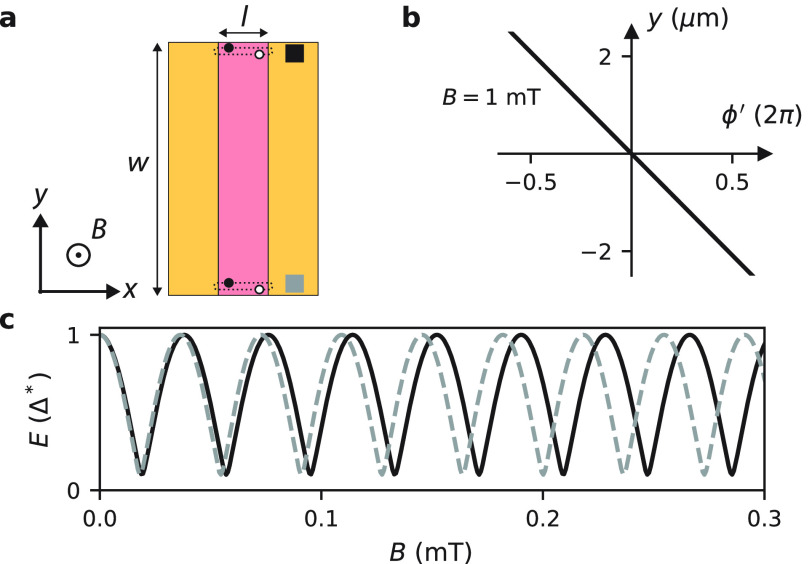
Effect of the magnetic vector potential. (a) Top-view schematic of the JJ in Dev 1. Two ABSs located at the top and bottom end are indicated. (b) Calculated local phase difference arising from the vector potential at *B* = 1 mT (*f* = 6.2). (c) Magnetic field evolution for the ABS located at the top (black) and bottom (gray), showing a relative shift due to the local phase difference.

With an understanding of the effect of the magnetic vector potential on the ABS spectrum, we now turn to spectroscopy measurements over a significantly larger field range (Figure 3). Figure 3a and b show the top and bottom spectroscopy maps, respectively. We first look at the high field regime (Figure 3a2 and b2), where the ABS oscillation amplitude has increased significantly (compare to 1b). This is caused by the Fraunhofer-like reduction of the critical current, *I*_c_, thereby reducing the so-called screening parameter, β ∝ *LI*_c_. The lower β results in a linear flux-phase relation, making it possible to probe the complete phase-dependence of the ABS (see SI-6 for more details). The fact that the ABS energy reaches very close to zero confirms that the ABSs we are probing have extremely high transparency.

**Figure 3:**
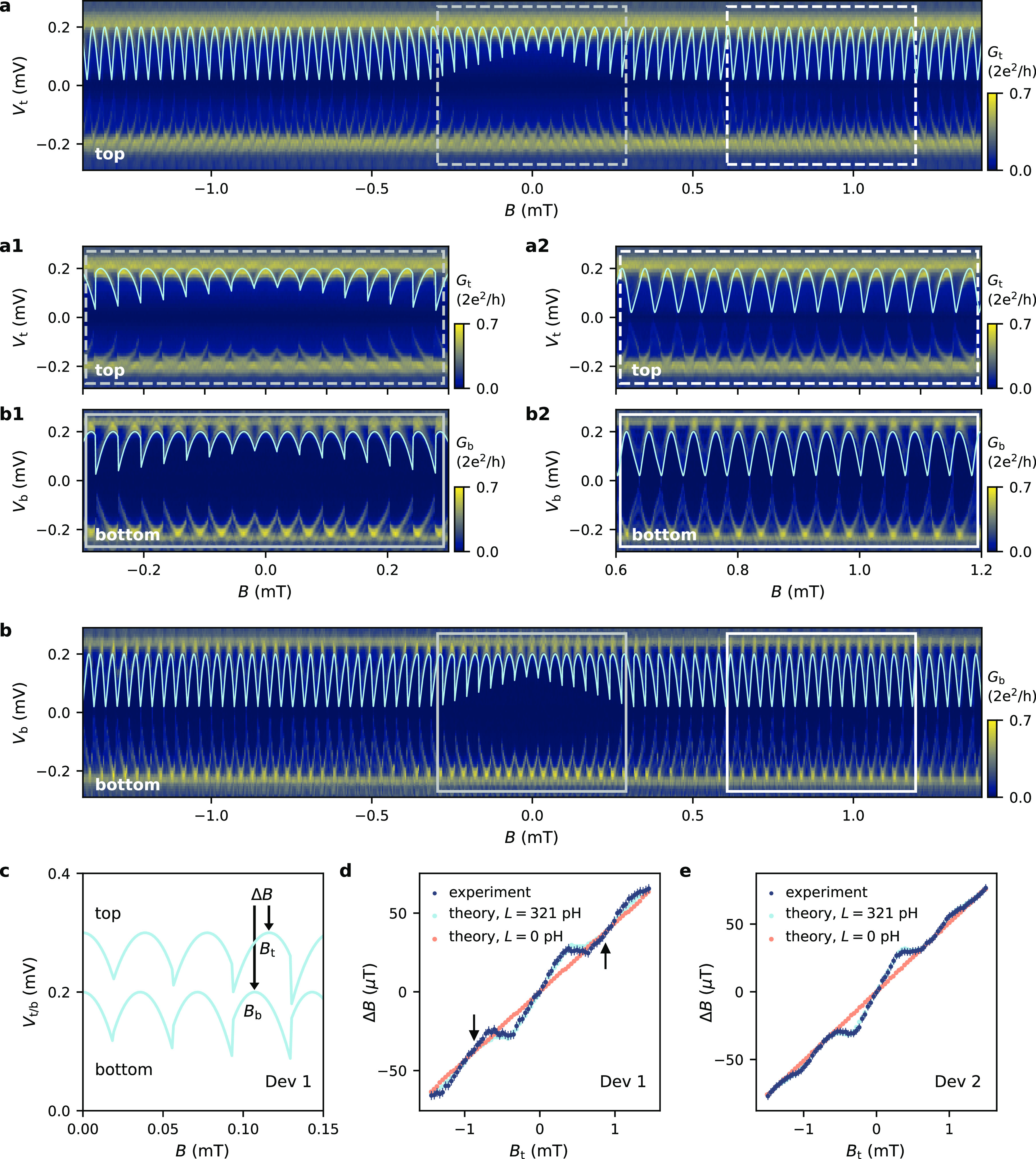
Tunneling spectroscopy over a large magnetic field range. (a) Spectroscopy map at the top end of Dev 1 (*V*_g1_ = −0.39 V, *V*_g2_ = −0.74 V, *V*_g3_ = 0 V, *V*_g4_ = 0 V), with zoomed-in views presented in panel a1 and a2. (b) Spectroscopy map at the bottom end (*V*_g1_ = 0 V, *V*_g2_ = 0 V, *V*_g3_ = −1.1 V, *V*_g4_ = −0.6 V) with zoom-in views in panel b1 and b2. The model (light blue lines) assumes coupling to a single ABS (τ = 0.99), taking into account the local phase difference in the JJ and the loop inductance (*L* = 321 pH). (c) Model curves for the top and bottom end plotted together (offsetted vertically for clarity). The ABS maxima on the top (*B*_t_) and bottom (*B*_t_) are shifted. (d,e) Δ*B* = *B*_t_ – *B*_b_ as a function of *B*_t_ for Dev 1 and Dev 2 (dark blue circles). We also include the Δ*B* values from the toy model with *L* = 321 pH (light blue circles), and *L* = 0 (red circles). The arrows indicate the position of the first Fraunhofer node.

In the intermediate field range (see Figure 3a1 and b1) we find that the cusps near the ABS minima develop into sharp jumps, resulting in a highly asymmetric and skewed shape away from *B* = 0. The skewness is not only reversed for positive and negative fields, but also for the top and bottom end of the JJ. Furthermore, we find that the ABS energy maxima shift in opposite directions in the top and bottom spectroscopy map, as expected for bound states localized at the edges. This is a strong indication that each probe is sensitive only to a region of limited spatial extent in its vicinity, and that it is in general difficult to reliably estimate bulk junction properties from a local spectroscopy measurement.^25^

To explain these findings we introduce a model that takes into account the combined effects of the inductance and vector potential, and assumes that each tunnel probe couples only to a single localized ABS with τ = 0.99 (a full description of the model can be found in SI-6). The resulting ABS spectra are shown as light blue lines plotted on the spectroscopy maps of Figure 3a and b. We find an excellent agreement between the model and the experiments in the entire magnetic field range. We show in SI-6 that the observed reversal of the skewness can only occur when both the vector potential and the loop inductance are taken into account. Therefore, the loop inductance serves as an extremely useful tool to clearly see the effects of a spatially varying phase difference along the JJ.

In order to systematically analyze the difference between the energy spectra of the top and bottom ABS, we introduce the quantity Δ*B* = *B*_t_ – *B*_b_ (see Figure 3c). In Figure 3d, we plot Δ*B* as a function of *B*_t_ for experiment (dark blue circles) and theory (light blue circles). Both show a nonlinear dependence, which can be well accounted for by the variation of *I*_c_ (and hence β). It is interesting to note that while our device geometry makes it impossible to directly measure *I*_c_ of the JJ, the nodes in the Fraunhofer pattern can still be identified by regions where β ≈ 0 (see arrows), and therefore the experiment/theory plots with finite *L* approach the theory curve with *L* = 0 (red circles). All of these findings are reproduced in Dev 2 (see spectroscopy maps in SI-5 and the Δ*B* analysis in Figure 3e).

Although our toy model is effective in capturing the most important features observed in experiments, it relies on the assumption that the tunnel probes couple to a single localized ABS in the vicinity of the barriers. In the following, we use numerical simulations to show that the tunneling current is indeed dominated by edge-located ABSs, and that the phase shifts for these states agree with the experiments. For the simulations, we consider a planar JJ composed of two semi-infinite superconducting leads and a normal region that is connected to two normal leads through tunneling barriers. We calculate the conductance from the top (bottom) normal lead, *G*_t_ (*G*_b_), by tracing the quasiparticles entering and leaving the top (bottom) lead. In the simulation, we include the effect of a perpendicular magnetic field and disorder, which results in a finite mean free path, *l*_e_. A superconducting phase difference, ϕ, is imposed between the superconducting terminals (more details about the model can be found in SI-7).

We first consider a ballistic JJ with infinite mean free path. In Figure 4a and b, we show the conductance calculated from the top and bottom, respectively, at *B* = 1 mT. In both maps, the main resonance is shifted by an equal amount in ϕ, but in opposite directions. This shift agrees very well with our toy model (black lines), where we assumed tunnel-coupling to a single ABS localized at the top/bottom end of JJ. The presence of localized ABSs is clearly seen by inspecting the supercurrent distribution calculated at the energy/phase values denoted by the colored circles in Figure 4a. We find that the top probe is only sensitive to the ABSs located in the vicinity of the top barrier (see Figure 4c).

**Figure 4:**
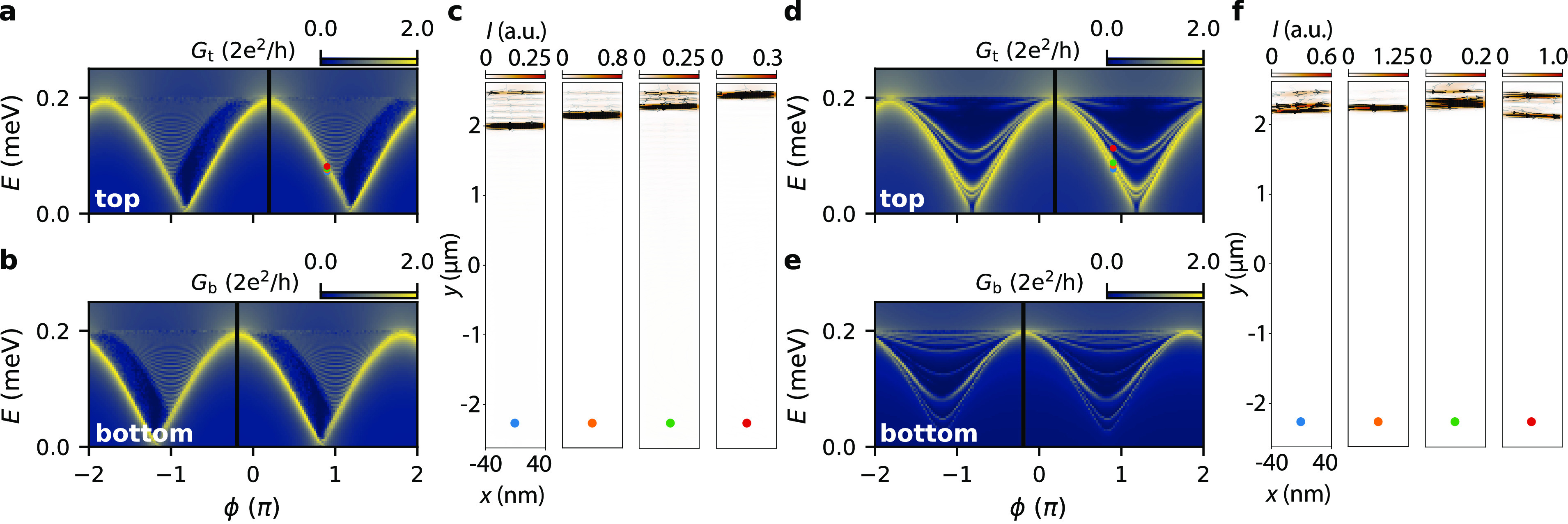
Numerical simulation of the tunneling conductance for a ballistic and disordered JJ. (a,b) Conductance maps at *B* = 1 mT for a ballistic JJ probed from the top and bottom. The black lines correspond to the phase shifts expected from the toy model. **c** Supercurrent distribution in the normal region of the JJ obtained for the *E* and ϕ values denoted with the circles in panel a. (d,e) Conductance maps at *B* = 1 mT for a disordered JJ (*l*_e_ = 150 nm) probed from the top and bottom. (f) Supercurrent distributions for the *E* and ϕ values denoted with the circles in panel d.

To make a connection with the experiments, we also consider a semiconductor with *l*_*e*_ = 150 nm, a good estimate for the mean free path in our 2DEGs.^22^ The top and bottom conductances are shown in 4d and e, respectively. As in the ballistic case, we again find a predominant sensitivity to edge-located ABSs, and a relative shift of the ABS maxima. However, we also note two important differences. First, unlike the ballistic case, the ABS spectra at the top and bottom are now drastically different from each other. This is not surprising, given the fact that the ABSs can be sensitive to the particular disorder configuration present at each end. Second, the main resonance splits into more clearly distinguishable ABSs. These ABSs are also localized close to the top/bottom end of JJ, as seen in Figure 4f. The specific location of these states is sensitive to the local potential landscape. However, we expect them to acquire different relative phase shifts depending on their precise location in the JJ.

This spatially dependent phase shift in the vicinity of the tunnel probe can also be experimentally observed. Figure 5a presents spectroscopy measurements on the top end of Dev 2, where the split gate settings have been modified to locally alter the disorder landscape. At *B* = 0 (central panel), distinct ABSs are hardly visible (see also black line cut in Figure 5b). However, when increasing the magnetic field (left and right panel), the localized ABSs acquire different phase shifts making it possible to resolve them more clearly (see also gray line cut in Figure 5b). Reversing the field direction leads to ABSs shifted in the opposite direction, as expected for spatially separated ABSs. A similar pattern of ABSs located at different positions close to the edge of the junction and experiencing different phase shifts is obtained in the numerical calculation shown in Figure 5c and d. This demonstrates that the effect of the vector potential (and resulting local phase difference) can indeed be used to estimate the location of the ABSs in the JJ. Around *B* = 2.09 mT, the maxima of the two states (indicated by the brown and pink circles) are shifted by ≈ 5 μT. This shift can be translated into an estimate of their spatial separation by using the spectroscopy results at the two extreme ends of the JJ (Figure S4 and Figure 3e), where we find Δ*B* = 106 μT at *B* = 2.09 mT for ABSs separated by 5 μm. Using this, we can estimate the spatial separation of the two states indicated by the brown and pink circles to be approximately 250 nm.

**Figure 5:**
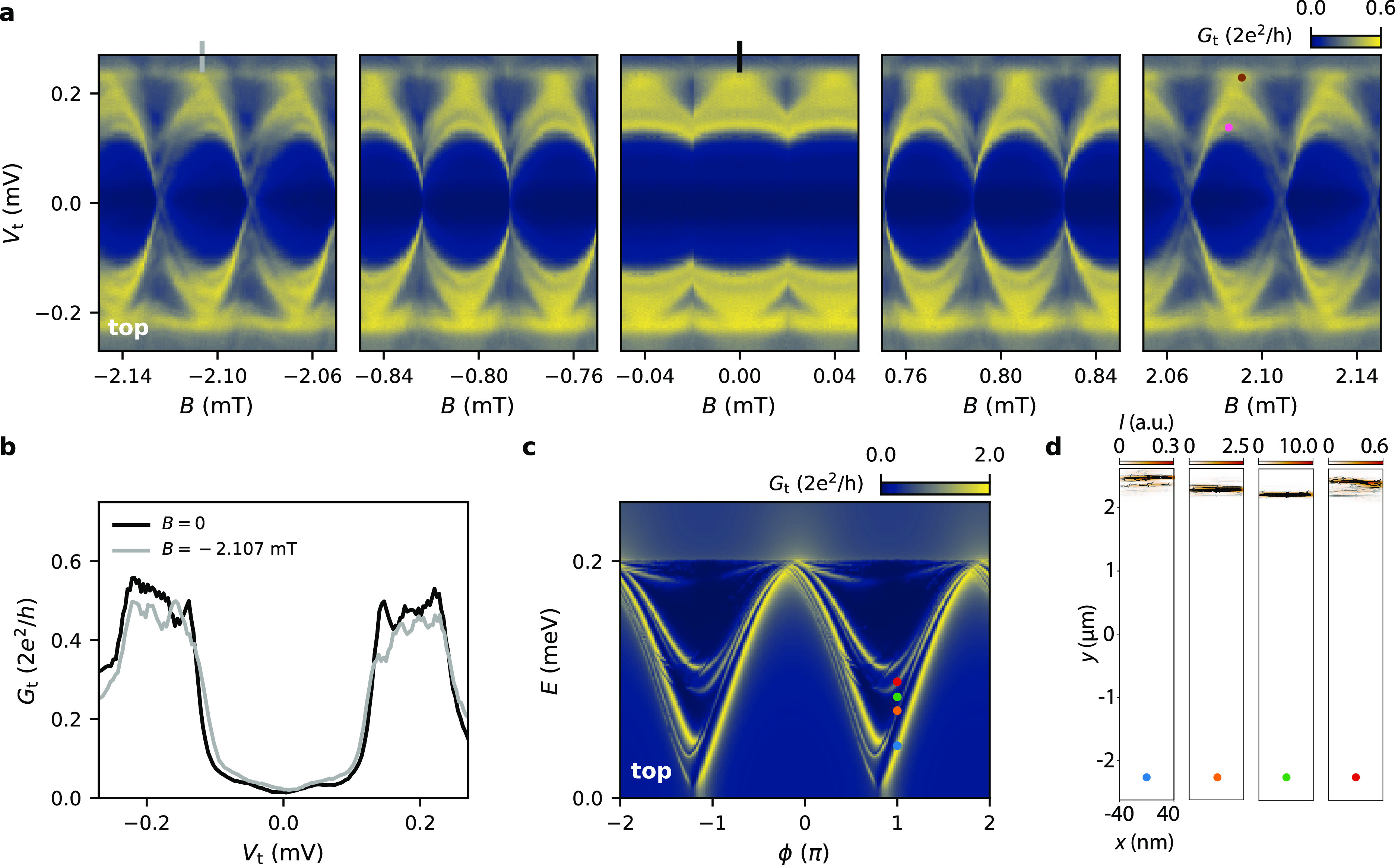
Probing spatially separated ABSs. (a) Tunneling spectroscopy maps at the top end of Dev 2 (*V*_g1_ = −1.90 V, *V*_g2_ = −1.40 V, *V*_g3_ = −2.10 V, *V*_g4_ = −1.43 V). The ABSs that are initially hardly resolvable around *B* = 0 are better resolved at larger *B*, where localized ABSs acquire different phase shifts depending on their location in the JJ. (b) Line cuts at two indicated positions in panel a showing this improvement in resolution. (c) Simulated tunneling conductance map for a disordered JJ (*l*_*e*_ = 150 nm) at *B* = 10 mT probed from the top. (d) Supercurrent distributions for the *E* and ϕ values marked by circles in panel c, showing how localized ABSs at different positions correspond to ABS spectra that are shifted in ϕ.

In conclusion, we employed local tunneling spectroscopy at two ends of planar phase-biased JJs to study the influence of the magnetic vector potential on the ABS spectrum. The combined effect of inductance and a spatially varying local phase difference results in striking differences in the tunneling spectra measured at the two edges of these junctions. Supporting our experiments with a theoretical toy model and microscopic numerical simulations, we showed that our results are consistent with the measurement of ABSs localized at the ends of the JJ, in the vicinity of the tunnel barriers. Finally, we showed that the effects of the vector potential are not only observable for ABSs separated by microns, but can also be used to estimate the relative locations of ABSs separated by a few hundred nanometers. Our results provide insights into the effects of a spatially varying phase difference on the ABS spectrum in extended JJs, and are relevant for ongoing efforts on investigating topological superconductivity in planar JJs.

Additional Note: During the preparation of this manuscript, we became aware of a related work on tunneling spectroscopy in planar JJs.^26^

## Notes

Raw data and analysis scripts for all presented figures are available at the 4TU.ResearchData repository: 10.4121/20059364.

## Supporting Information Available

Device fabrication, estimation of loop inductance, flux focusing in planar JJ, tunneling spectroscopy for Dev 2, toy model, microscopic model (PDF)
