# Supplementary material for: Controlling Andreev Bound States with the Magnetic Vector Potential
Source: Nano Lett. 2022 Oct 24;22(21):8601–7. doi: 10.1021/acs.nanolett.2c03130 (PMC9650727; doi:10.1021/acs.nanolett.2c03130)
Supplement: Supplementary file 1 — nl2c03130_si_001.pdf [file nl2c03130_si_001.pdf]

Supplementary Information for: Controlling Andreev bound states with the magnetic vector potential

Christian M. Moehle,[1,*] Prasanna K. Rout,[1,*] Nayan A. Jainandunsing,[1]
Dibyendu Kuiri,[2] Chung Ting Ke,[1,†] Di Xiao,[3] Candice Thomas,[3]
Michael J. Manfra,[3,4,5,6] Michał P. Nowak,[2] and Srijit Goswami[1]

[1]*QuTech and Kavli Institute of Nanoscience, Delft University of Technology, 2600 GA Delft, The Netherlands*

[2]*AGH University of Science and Technology, Academic Centre for Materials and Nanotechnology, 30-059 Krakow, Poland*

[3]*Department of Physics and Astronomy, Purdue University, West Lafayette, Indiana 47907, USA*

[4]*Elmore School of Electrical and Computer Engineering, Purdue University, West Lafayette, Indiana 47907, USA*

[5]*School of Materials Engineering, Purdue University, West Lafayette, Indiana 47907, USA*

[6]*Microsoft Quantum Lab West Lafayette, West Lafayette, Indiana 47907, USA*

1. DEVICE FABRICATION

The two phase-biased JJs (Dev 1, Dev 2) and the DC SQUID are fabricated using electron beam lithography. Due to a possible intermixing of Al and Sb we perform all fabrication steps at room temperature unless stated otherwise. The device fabrication starts by etching the Al and the 2DEG in unwanted areas. The Al etch is performed in Transene D etchant at a temperature of 48.2 °C for 9 s resulting a etching thickness of 100 nm. Afterwards, using the same PMMA mask, the 2DEG is etched in a solution of 560 ml deionized water, 9.6 g citric acid powder, 5 ml H_2O_2 and 4 ml H_3PO_4, using an etching time of 90 s. To define the JJs, we perform a second Al etch, carried out in 38.2 °C Transene D for 16 s. This is followed by sputtering a 60 nm thick layer of SiN_x that partly covers the superconducting loop, isolating it from the intended 2DEG contact inside the loop. Next, we contact the exposed 2DEG region on the top and bottom side of the JJ by Ti/Au. Prior to the evaporation of 10 nm Ti and 190 nm Au, a gentle Ar etching is performed in the loadlock of the evaporator to remove any oxides that might have formed on the 2DEG. Afterwards, we contact the superconducting loop by sputtering 150 nm of NbTiN (before the sputter process an in-situ Ar etch is performed to remove the oxide on the Al). As the gate dielectric, we deposit a global layer (40 nm thick) of AlO_x by atomic layer deposition at 40 °C. The gates are formed in two steps: First, the fine structures (split gates and central gate) are deposited by evaporating 10 nm of Ti and 40 nm of Au. In the second step, 10 nm Ti and 100 nm Au are evaporated to define the gate leads.

A schematic and false-colored SEM of Dev 1 is shown in Fig. 1a of the main text. In Fig. S1a we present a SEM of Dev 2, which is similar to Dev 1. The main difference is that the normal region of the JJ is slightly zigzag-shaped ($z_x = 0.24\,\mu$m, $z_y = 1.43\,\mu$m). This was originally introduced into this device to potentially suppress long quasiparticle trajectories and thereby increase the size of the topological gap [1]. The superconducting leads of Dev 1 and Dev 2 have a length of 500 nm. Figure S1b shows a SEM of the DC SQUID, consisting of two JJs (device JJ and reference JJ) in the superconducting loop. The device JJ has a superconducting lead length of 300 nm. Two additional gates are deposited, one covering the normal region of the reference JJ and one covering the 2DEG region around this junction

* These authors contributed equally to this work.
† Present Address: Institute of Physics, Academia Sinica, Taipei, 11529, Taiwan

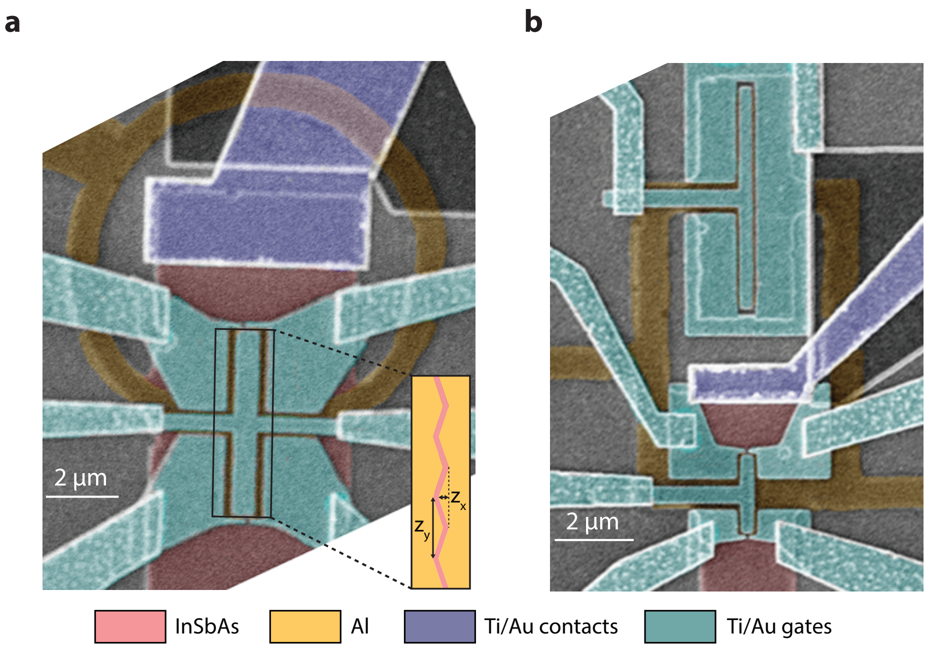

FIG. S1. **a** SEM of Dev 2 having a zigzag-shaped normal region ($z_x = 0.24\,\mu$m, $z_y = 1.43\,\mu$m) with a length of $l = 80\,$nm and width of $w = 5\,\mu$m. **b** SEM of the DC SQUID. The device JJ (on the bottom) has dimensions $l = 120\,$nm and $w = 2\,\mu$m. The reference JJ (on the top) has dimensions $l = 80\,$nm and $w = 5\,\mu$m.

(always kept at -2.5 V to deplete the 2DEG there).

2. ESTIMATION OF LOOP INDUCTANCE

In order to extract the inductance of the SQUID loop, we measure the SQUID interference pattern for different reference JJ gate voltages, $V_{\mathrm{g,ref}}$. Figure S2a-l shows the obtained differential resistance maps as a function of applied current bias, I, and perpendicular magnetic field, B. Panel a-l corresponds to $V_{\mathrm{g,ref}}$ = 0, -0.4, -0.8, -0.9, -1, -1.1, -1.2, -1.25, -1.3, -1.35, -1.4, and -1.45 V, respectively. The device JJ gate is grounded in all measurements. With the colored circles we mark the positions where the total critical current is maximum. For a given SQUID oscillation, the field at which the maximum occurs is different for positive and negative current bias: $\Delta B = B^+ - B^-$. The corresponding flux difference is given by: $\Delta\Phi = 2(L_{\mathrm{ref}} I_{\mathrm{c,ref}} - L_{\mathrm{dev}} I_{\mathrm{c,dev}})$ [2]. Here, $I_{\mathrm{c,ref}}$ and $I_{\mathrm{c,dev}}$ are the critical current of the reference and device junction, respectively. The inductances of the two SQUID arms are

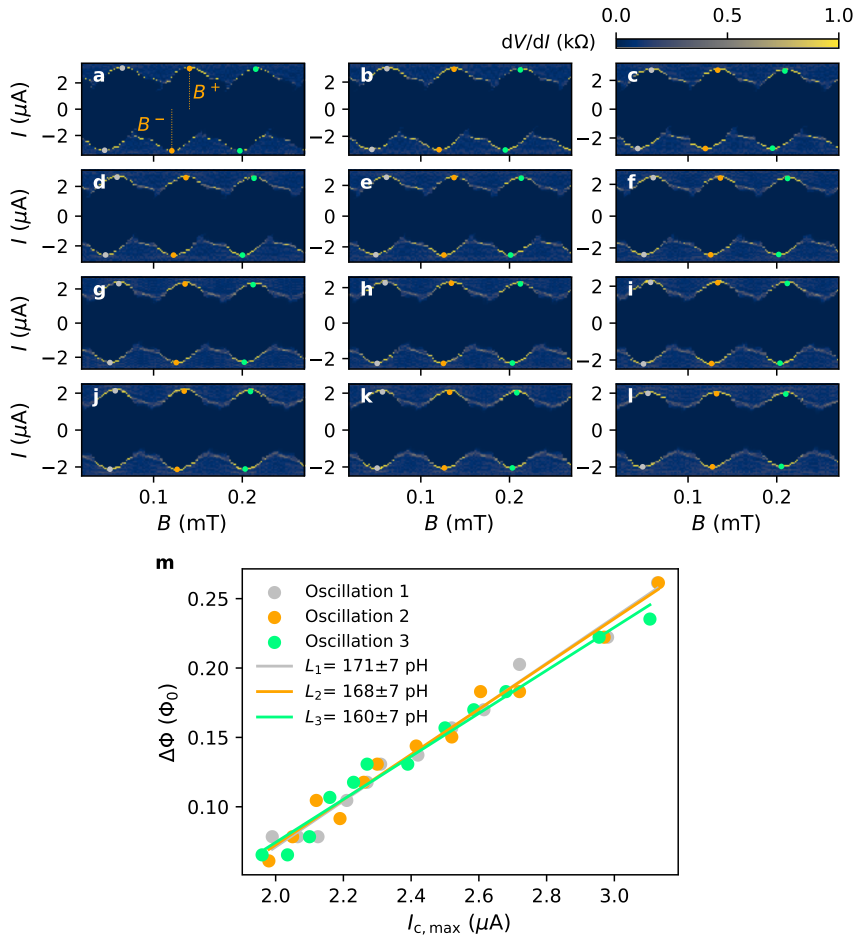

FIG. S2. Differential resistance, $\mathrm{d}V/\mathrm{d}I$, as a function of applied current bias, I, and perpendicular magnetic field, B. Panel **a-l** corresponds to reference gate voltage $V_{\mathrm{g,ref}} = 0$, -0.4, -0.8, -0.9, -1, -1.1, -1.2, -1.25, -1.3, -1.35, -1.4, and -1.45 V, respectively. No voltage is applied to the device junction gate. The colored circles mark the positions of maximum total critical current. **m** $\Delta\Phi$ plotted against $I_{\mathrm{c,max}}$ for the three oscillations shown in **a-l**. The extracted ΔB is normalized with respect to the oscillation period, giving $\Delta\Phi$ in units of the magnetic flux quantum, Φ_0. The average value of the maximum critical current on the positive and negative current bias sides gives $I_{\mathrm{c,max}}$.

L_{ref} and L_{dev}. The above expression can be rewritten as: $\Delta\Phi = 2L_{\mathrm{ref}}I_{\mathrm{c,max}} - 2LI_{\mathrm{c,dev}}$, using the relations for the maximum critical current, $I_{\mathrm{c,max}} = I_{\mathrm{c,ref}} + I_{\mathrm{c,dev}}$, and the total loop inductance, $L = L_{\mathrm{ref}} + L_{\mathrm{dev}}$.

In Fig. S2m we plot the extracted $\Delta\Phi$ as a function of $I_{\mathrm{c,max}}$ for the three oscillations indicated in Fig. S2a-l. The linear fits yield $L_{\mathrm{ref}} = 166$ pH as the average value. Since the width

and the thickness of the superconducting loop is the same for all three devices, the inductance should only depend on the length of the superconducting loop. Under this assumption the loop inductance of the phase-biased JJs can be estimated to be $L_{\mathrm{ref}} l_{\mathrm{PBJJ}}/l_{\mathrm{ref}} = 321\,\mathrm{pH}$, where $l_{\mathrm{ref}} = 15.3\,\mu\mathrm{m}$ is the length of SQUID reference arm and $l_{\mathrm{PBJJ}} = 29.6\,\mu\mathrm{m}$ is the loop length of Dev 1 and 2.

3. FLUX FOCUSING IN PLANAR JJ

The Fraunhofer interference pattern periodicity, B_0, in a JJ is determined by the geometrical area, A, enclosed between two superconducting leads, i.e. $B_0 = \Phi_0/A$. However, in the presence of flux focusing, the periodicity is reduced from the theoretical value [3]. To estimate the effect of flux focusing we measure the differential resistance, $\mathrm{d}V/\mathrm{d}I$, as a function of applied current, I, and perpendicular magnetic field, B, for the device JJ of the DC SQUID (see Fig. S3). For this measurement, the reference JJ is pinched off by applying a voltage of $-2.5\,\mathrm{V}$ to the top gate. We observe the first node at $2.1\,\mathrm{mT}$ instead of the expected Fraunhofer periodicity of $B_0 = 8.6\,\mathrm{mT}$. This gives a dimensionless flux focusing factor, f, of 4.1 for this junction.

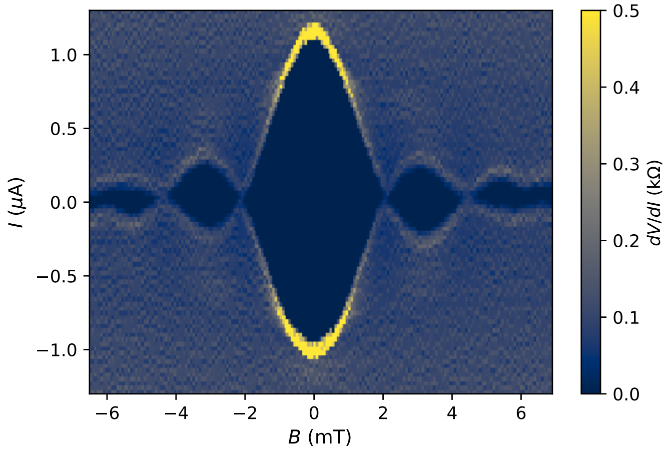

FIG. S3. Differential resistance, $\mathrm{d}V/\mathrm{d}I$, as a function of applied current, I, and perpendicular magnetic field, B for the device JJ of the DC SQUID.

To explain our spectroscopy maps measured at the top and bottom ends of Dev 1 and Dev 2 we introduce a toy model with flux focusing in Sec. 6. Although the above extracted f

gives an estimate of the focusing factor, the exact value can vary from junction to junction. The best agreement between the experimental spectroscopy maps and the toy model is be achieved with $f = 6.2$ for Dev 1 and 7.2 for Dev 2 (see Fig. 3 in the main text as well as Fig. S4]. The larger f values (and therefore stronger flux focusing) are in fact expected due to the shorter JJ length and larger lead length of Dev 1 and Dev 2 compared to the values for the device JJ of the DC SQUID [3].

4. JOSEPHSON PENETRATION DEPTH

The Josephson penetration depth for a JJ with the thickness of the superconducting electrodes comparable or smaller than the penetration depth is dominated by the kinetic inductance contribution and is given as [4]: $\lambda_J = (\Phi_0 w/4\pi\mu_0 J_c \lambda^2)^{1/2}$, where $w = 5\,\mu$m is the junction width, J_c is the critical current density, and λ is the superconducting penetration depth of Al.

For our junctions, the thickness of the Al electrodes (7 nm) is much smaller compared to the previously reported value of $\lambda = 180$ nm for a similar heterostructure [3]. Therefore we use the above expression to determine λ_J. Since the critical current cannot be measured for Dev 1 and Dev 2, we estimate it based on values obtained for the DC SQUID. The critical current of the device JJ with width $w = 2\,\mu$m is $I_c = 1.05\,\mu$A (see Fig. S3) and the thickness of 2DEG is $t = 30\,$nm. Using these values we get $J_c = I_c/wt = 1.75 \times 10^7 \text{A/m}^2$ and $\lambda_J = 34\,\mu$m, which is much larger than the width of the JJs ($w = 5\,\mu$m). This ensures that the gauge-invariant phase difference can be expressed as $\varphi(y) = \phi + \phi'$, with $\phi' = -2\pi\frac{fBly}{\Phi_0}$.

5. TUNNELING SPECTROSCOPY FOR DEV 2

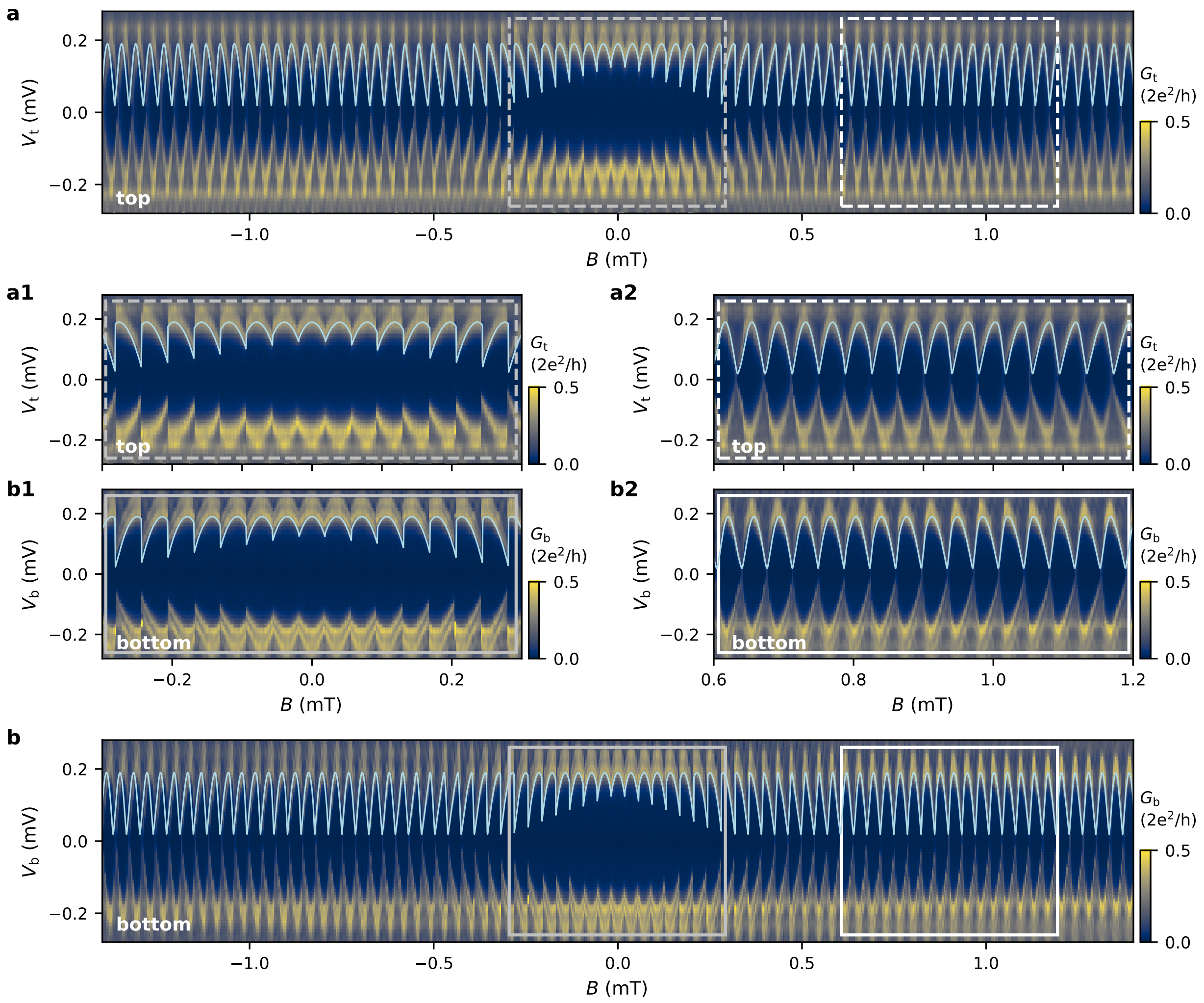

FIG. S4. **a** Spectroscopy map at the top end of Dev 2 with zoom-ins presented in **a1** and **a2**. The bottom spectroscopy map is shown in **b** with zoom-ins in **b1** and **b2**. Both measurements were obtained with $V_{g1} = -1.60\,\mathrm{V}$, $V_{g2} = -1.42\,\mathrm{V}$, $V_{g3} = -2.10\,\mathrm{V}$, $V_{g4} = -1.43\,\mathrm{V}$. The model (light blue line) assumes coupling to a single ABS ($\tau = 0.99$), taking into account the local phase difference in the JJ and the loop inductance ($L = 321\,\mathrm{pH}$) for the field-phase conversion.

6. TOY MODEL

This model is used to calculate the Andreev bound states (ABSs) energies of a Josephson junction embedded in a superconducting loop in the presence of a perpendicular magnetic field, as used to substantiate the measurement results shown in Fig. 3 and Fig. S5. The junction has a length l (the distance between the superconducting contacts) and a width w (the distance between the edges of the junction where the tunneling probes are connected).

We assume a homogeneous density of the supercurrent in the junction and that the current is carried by M ABSs uniformly distributed across the junction at positions $y_n = -w/2 + (n-1) \cdot w/(M-1)$ with integer $n \in [1, M]$.

The positive energies of the ABSs in the junction with the transmission coefficient τ are given by [5]:

$$E_n(\varphi_n) = \Delta\sqrt{1 - \tau \sin^2(\frac{\varphi_n}{2})}, \tag{S1}$$

where, in the presence of the external perpendicular magnetic field, $\varphi_n = \phi + \frac{2\pi}{\Phi_0} \int_{(0,y_n)}^{(l,y_n)} \mathbf{A} \cdot d\mathbf{l}$ is the gauge-invariant phase drop across the junction for an ABS located at position y_n. ϕ is the superconducting phase difference. For the vector potential in the Landau gauge $\mathbf{A} = (-yB, 0, 0)$, the phase drop in the junction at y_n is $\varphi_n = \phi - (2\pi/\Phi_0) \cdot fBly_n$, where we included f as the magnetic field focusing factor. The latter equation gives the phase evolution of the ABS located at the edges of the junction as $\varphi_{\mathrm{t/b}} = \phi \mp (\pi/\Phi_0) \cdot fBlw$ [6] with a minus (plus) sign for the upper (bottom) edge.

The zero-temperature supercurrent obtained from the positive-energy ABSs in the junction is given by:

$$I(\varphi) = \frac{e\Delta^2\tau}{2\hbar} \sum_{n}^{M} \frac{\sin(\varphi_n)}{E_n(\varphi_n)}. \tag{S2}$$

In the experimental setup, the superconducting phase difference ϕ is induced by a flux $\Phi = B\pi R^2$ that threads a superconducting loop with radius R. The non-zero loop inductance L results in the following phase-flux relation [7]:

$$\phi = \frac{2\pi}{\Phi_0}(\Phi - LI(\varphi)). \tag{S3}$$

We obtain the energies of the ABSs located at the edges of the junction versus B using the following procedure. In the first step, we solve Eq. S3 for a given B and obtain the ϕ value that minimizes the total energy of the system $E(\phi) = LI^2(\varphi)/2 - \sum_n^M E_n(\varphi_n)$ calculated

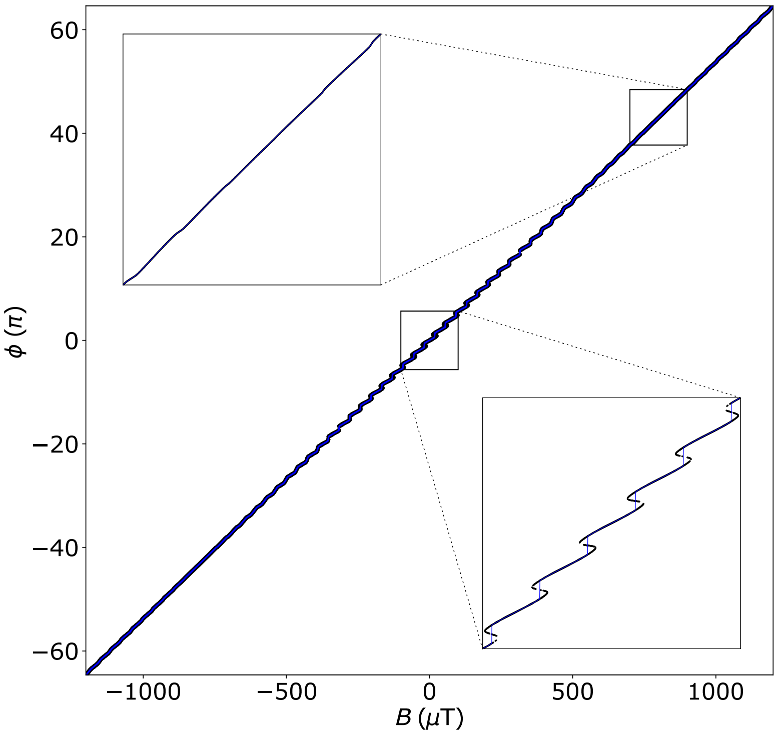

FIG. S5. Superconducting phase difference versus applied magnetic field obtained for $L = 321$ pH, $l = 80$ nm, $w = 5000$ nm, $M = 35$, $\tau = 0.99$, $R = 4207$ nm, $\Delta = 0.2$ meV and $f = 6.2$. The black dots show possible phase values for a given B, while the blue curve shows the superconducting phase difference obtained by minimizing the total energy.

as the sum of the energy contained in the superconducting loop and the free energy of the junction ($F = const - E_j = const - \sum E_n$). An example of a flux-to-phase conversion curve is shown in Fig. S5. Finally, we use the phase difference value to calculate E_n corresponding to the ABSs located at the outermost edges of the junction using Eq. S1.

Figure S6 shows an ABS located at the top (top panel) and bottom (bottom panel) end of the JJ in the presence and absence of the loop inductance and the local phase difference arising from the magnetic vector potential as indicated. The reversal of the skewness can only happen when both the loop inductance and the local phase difference are present.

Table S1 summarizes the parameters that are used for the overlays for Dev 1 (Fig. 3 of the main text) and Dev 2 (Fig. S4).

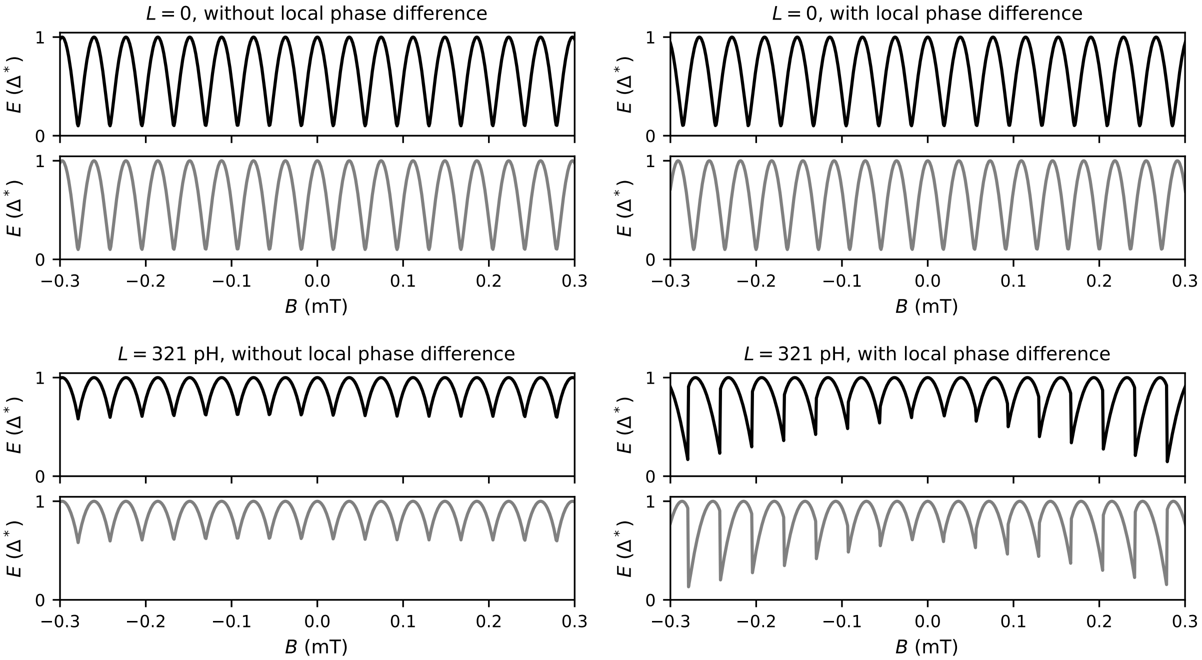

FIG. S6. ABS energy versus magnetic field in the presence or absence of the loop inductance and with or without local phase difference in the JJ as indicated. The top and bottom panels correspond to an ABS located at the top and bottom end of the JJ, respectively. For all plots, the following parameters are used: $l = 80$ nm, $w = 5000$ nm, $M = 35$, $\tau = 0.99$, $R = 4207$ nm, $\Delta = 0.2$ meV and $f = 6.2$.

Parameter	Dev 1	Dev 2
l (nm)	80	80
w (μm)	5	5
R (nm)	4207	4190
L (pH)	321	321
Δ (meV)	0.2	0.19
f	6.2	7.2
M	35	45
τ	0.99	0.99

TABLE S1. Toy model parameters used for Dev 1 and Dev 2.

7. MICROSCOPIC MODEL

A. Tunneling spectroscopy calculations

We consider a four-terminal device, with two vertical superconducting leads separated by the normal region (which creates the superconductor-normal-superconductor junction) and two normal leads that are placed horizontally at the top and bottom—see Fig. S7. Between the horizontal leads and the normal scattering region, we introduce tunneling barriers that mimic the behavior of QPCs tuned into the tunneling regime.

The considered system is described by the Hamiltonian

$$\begin{bmatrix} H & \Delta \\ \Delta^* & -H \end{bmatrix}, \tag{S4}$$

acting on a wave function in the basis $\Psi = (\Psi_e, \Psi_h)^T$. Here H is defined as

$$H = -\frac{\hbar^2}{2m^*}\nabla^2 + V(r) - \mu. \tag{S5}$$

μ is the chemical potential, m^* is the effective electron mass and $V(r)$ is the rectangular potential barrier of height V_g placed just above and below the normal region of length ($l = 80$ nm).

In the presence of a magnetic field, the Hamiltonian H becomes

$$H^{'} = -\frac{\hbar^2}{2m^*}(\nabla - q\mathbf{A}/\hbar)^2 + V(r) - \mu, \tag{S6}$$

with $q = -|e|$ for the electron and $q = |e|$ for the hole part of the Hamiltonian Eq. S4. We choose the vector potential in the Landau gauge with $\vec{B} = B\hat{z}$, so that $\vec{A} = -By\hat{x}$

The superconducting pairing potential Δ varies spatially and is given by:

$$\Delta(x) = \begin{cases} \Delta_0 & \text{if } x < -l/2 \\ 0 & \text{if } -l/2 \leq x \leq l/2 \\ \Delta_0 e^{\iota\phi} & \text{if } x > l/2 \end{cases}$$

We discretize the Hamiltonian Eq. S4 on a square lattice with discretization constant $a = 10$ nm. We put the material parameters as $m^* = 0.016m_e$, $\mu = 5$ meV, $\Delta = 0.2$ meV. We introduce the anisotropic mass in the superconducting leads with the mass along the

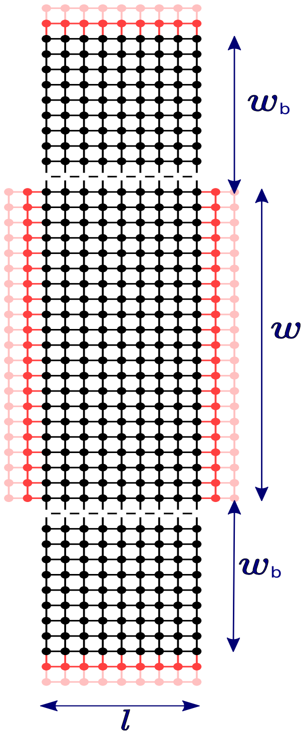

FIG. S7. Schematic of the system considered for tunneling spectroscopy calculations. The dots denote the sites of the computational mesh. The black dots correspond to the scattering region, whereas the pink ones denote the semi-infinite leads. We use $l = 80$ nm (the distance between the superconducting contacts) and $w = 5000$ nm (the distance between the edges of the junction where the tunneling probes are connected). The barrier potential at the top and bottom is separated from the normal leads of width $w_b = 100$ nm. The vertical leads are superconducting, while the horizontal leads are normal.

translation symmetry of the superconducting leads equal to $10m^*$ as appropriate for the description of transparent normal-superconductor interfaces in models where the chemical potential is kept constant [8]. Including a vector potential in this system is done using Peierls substitution as $t_{nm} \rightarrow t_{nm} \exp[\frac{-\imath e}{\hbar} \int \mathbf{A} d\mathbf{l}]$ [9, 10].

We exclude the magnetic field from the superconducting leads to account for the screening effect setting $\mathbf{A} = 0$ there. We also put zero vector potential in the top and bottom leads to maintain the translation invariance. This in turn introduces a delta peak in the magnetic field where the horizontal leads are attached (as calculated from $\mathbf{B} = \nabla \times \mathbf{A}$). We have,

however, verified that for the considered small magnetic fields, this does not affect our results, as confirmed by replacing the vector potential by a position-dependent superconducting phase as $\phi \to \phi - \frac{2\pi Bly}{\Phi_0}$ and observing that both results match accurately.

The finite mean free path (l_e) is implemented by introducing a random on-site disorder potential $V_d(x, y)$ with the amplitude uniformly distributed between $-U_d/2$ and $U_d/2$ [11], where

$$U_d = \mu\sqrt{\frac{6\lambda_F^3}{\pi^3 a^2 l_e}}. \tag{S7}$$

Here a, l_e, λ_F are the lattice constant, mean free path and the Fermi wavelength, respectively. We calculate the conductance map with respect to the phase difference ϕ and energy using the scattering matrix approach implemented in the Kwant package [12], using the formula:

$$G_{t/b} = \frac{2e^2}{h}(N^e_{t/b} - T^{ee}_{t/b} + T^{he}_{t/b}), \tag{S8}$$

where t and b stand for top and bottom lead respectively and $N^e_{t/b}$ is the corresponding number of the electron modes. The energy dependent transmissions are evaluated as:

$$T^{\alpha\beta}_{t/b} = Tr([S^{\alpha\beta}_{t/b}]^\dagger S^{\alpha\beta}_{t/b}), \tag{S9}$$

where $S^{\alpha\beta}_{t/b}$ is the block of scattering amplitudes of incident particle of type β in t (b) lead to a particle of type α in the lead t (b).

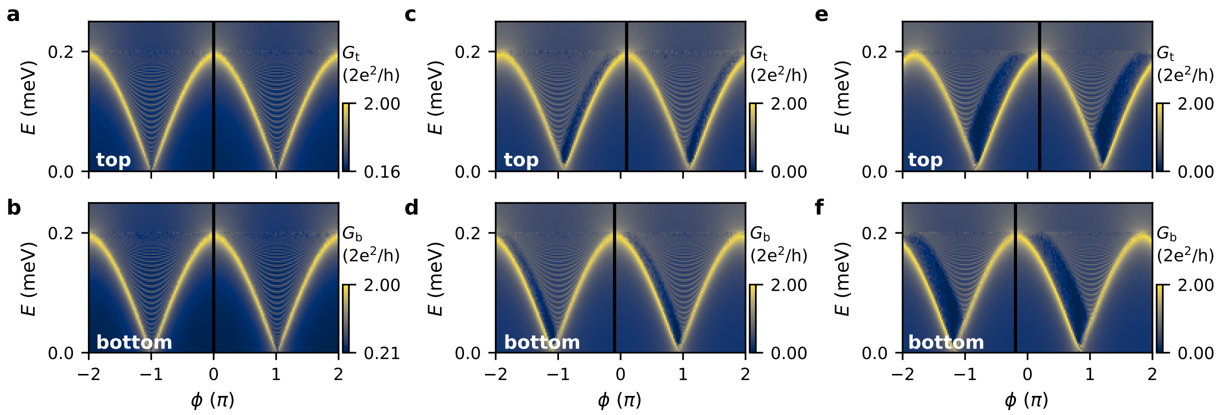

FIG. S8. Conductance versus phase difference and energy calculated for quasiparticles injected from the top lead (upper row) and bottom lead (lower row) at $B = 0$ (**a**,**b**), $B = 0.5$ mT (**c**,**d**) and $B = 1$ mT (**e**,**f**). The vertical black lines denote the expected phase shift of the edge modes due to the magnetic field $\varphi_{\mathrm{t/b}} = \phi \mp (\pi/\Phi_0) \cdot fBlw$.

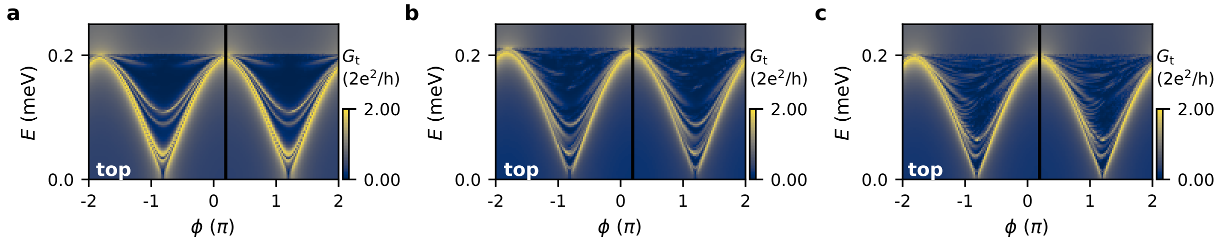

FIG. S9. Conductance versus phase difference and energy calculated for quasiparticles injected from the top lead for $B = 1$ mT and different mean free paths $l_e = 150$ **a**, 500 **b** and 1000 nm **c**.

B. ABS calculation

For the numerical calculation of ABSs spectra we consider a Josephson junction treated as a finite system consisting of a normal scattering region and two long superconducting segments. The two superconducting regions have a length of $l_{SC} = 2000$ nm (much larger than the coherence length $\xi = 1091.16$ nm, calculated using the formula, $\xi = \frac{\hbar v_F}{\Delta}$ where $v_F = \sqrt{2\mu/m^*}$), and they are separated by a normal region of length $l = 80$ nm. The width w of the entire system is taken as 1000 nm. The Hamiltonian remains the same as in equation S6 except for the tunneling barrier potentials (here we do not consider the top and bottom electrode). The anisotropic mass and Peierls phase factor (for magnetic vector potential) are introduced as described above. We diagonalize the Hamiltonian and plot the energy with respect to the phase difference ϕ, and also the probability current in Fig. S10. In the probability current, we observe that in the presence of the perpendicular field each ABSs is localized in a separated region in the junction. The different spatial position of the ABSs is reflected by their different phase shifts in the spectrum plotted in Fig. S10 (a).

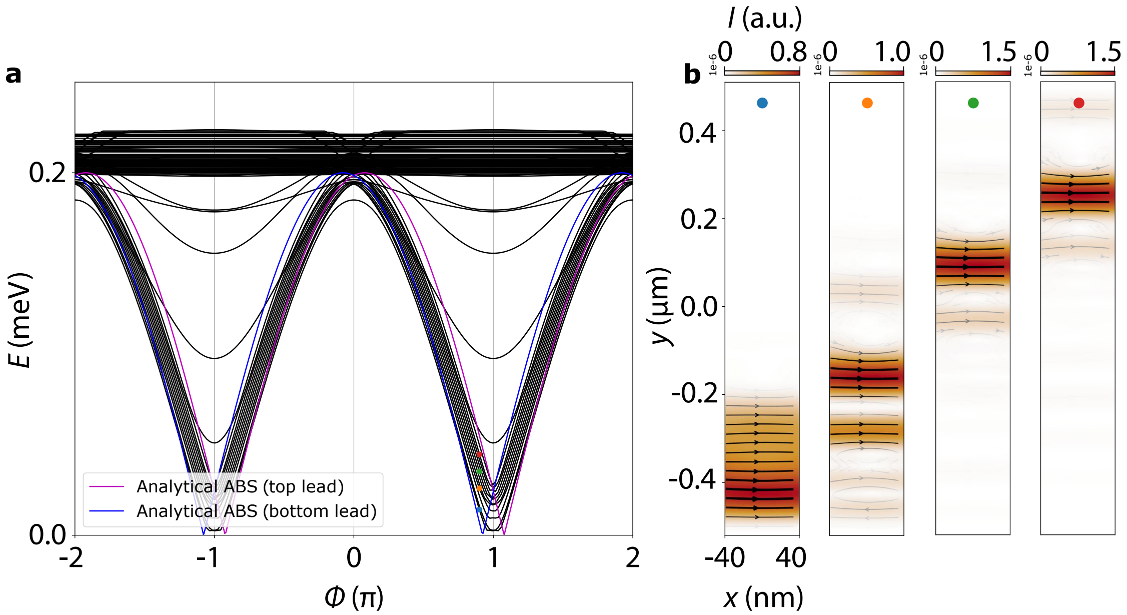

FIG. S10. **a** ABS spectrum of a SNS junction with two SC regions ($l_{SC} = 2000$ nm) separated by a normal region ($l = 80$ nm, $w = 1000$ nm) at $B = 2$ mT without disorder. The color curves denote analytically calculated ABS from Eq. S1 with $\tau = 1$. **b** Supercurrent in the normal area of the junction calculated for the ABS whose energies are denoted by the color circles in **a**.

References

---

[1] T. Laeven, B. Nijholt, M. Wimmer, and A. R. Akhmerov, Enhanced proximity effect in zigzag-shaped majorana josephson junctions, Phys. Rev. Lett. **125**, 086802 (2020).

[2] A. I. Braginski and J. Clarke, *The SQUID Handbook* (John Wiley and Sons, Ltd, 2004).

[3] H. J. Suominen, J. Danon, M. Kjaergaard, K. Flensberg, J. Shabani, C. J. Palmstrøm, F. Nichele, and C. M. Marcus, Anomalous Fraunhofer interference in epitaxial superconductor-semiconductor Josephson junctions, Phys. Rev. B **95**, 035307 (2017).

[4] S. K. Tolpygo and M. Gurvitch, Critical currents and Josephson penetration depth in planar thin-film high-t_c Josephson junctions, Appl. Phys. Lett. **69**, 3914 (1996).

[5] C. W. J. Beenakker, Universal limit of critical-current fluctuations in mesoscopic Josephson junctions, Phys. Rev. Lett. **67**, 3836 (1991).

[6] D. Sticlet, P. Wójcik, and M. P. Nowak, Squid pattern disruption in transition metal dichalcogenide Josephson junctions due to nonparabolic dispersion of the edge states, Phys. Rev. B **102**, 165407 (2020).

[7] A. Banerjee, O. Lesser, M. A. Rahman, H. R. Wang, M. R. Li, A. Kringhøj, A. M. Whiticar, A. C. C. Drachmann, C. Thomas, T. Wang, M. J. Manfra, E. Berg, Y. Oreg, A. Stern, and C. M. Marcus, Signatures of a topological phase transition in a planar Josephson junction, arXiv.2201.03453 (2022).

[8] D. Sticlet, B. Nijholt, and A. Akhmerov, Robustness of Majorana bound states in the short-junction limit, Phys. Rev. B **95**, 115421 (2017).

[9] R. Peierls, Zur theorie des diamagnetismus von leitungselektronen, Zeitschrift für Physik **80**, 763 (1933).

[10] D. R. Hofstadter, Energy levels and wave functions of bloch electrons in rational and irrational magnetic fields, Phys. Rev. B **14**, 2239 (1976).

[11] T. Ando, Quantum point contacts in magnetic fields, Phys. Rev. B **44**, 8017 (1991).

[12] C. W. Groth, M. Wimmer, A. R. Akhmerov, and X. Waintal, Kwant: a software package for quantum transport, New J. Phys. **16**, 063065 (2014).
